# Molecular links between cellular senescence, longevity and age-related diseases – a systems biology perspective

**DOI:** 10.18632/aging.100413

**Published:** 2011-12-18

**Authors:** Robi Tacutu, Arie Budovsky, Hagai Yanai, Vadim E. Fraifeld

**Affiliations:** ^1^ The Shraga Segal Department of Microbiology and Immunology, Center for Multidisciplinary Research on Aging, Ben-Gurion University of the Negev, Beer Sheva, Israel; ^2^ The Judea Regional R&D Center, Moshav Carmel, Israel

**Keywords:** cellular senescence, age-related diseases, genes, microRNAs, pathways, networks

## Abstract

The role of cellular senescence (CS) in age-related diseases (ARDs) is a quickly emerging topic in aging research. Our comprehensive data mining revealed over 250 genes tightly associated with CS. Using systems biology tools, we found that CS is closely interconnected with aging, longevity and ARDs, either by sharing common genes and regulators or by protein-protein interactions and eventually by common signaling pathways. The most enriched pathways across CS, ARDs and aging-associated conditions (oxidative stress and chronic inflammation) are growth-promoting pathways and the pathways responsible for cell-extracellular matrix interactions and stress response. Of note, the patterns of evolutionary conservation of CS and cancer genes showed a high degree of similarity, suggesting the co-evolution of these two phenomena. Moreover, cancer genes and microRNAs seem to stand at the crossroad between CS and ARDs. Our analysis also provides the basis for new predictions: the genes common to both cancer and other ARD(s) are highly likely candidates to be involved in CS and *vice versa*. Altogether, this study shows that there are multiple links between CS, aging, longevity and ARDs, suggesting a common molecular basis for all these conditions. Modulating CS may represent a potential pro-longevity and anti-ARDs therapeutic strategy.

## INTRODUCTION

Since Hayflick's discovery of the phenomenon of cellular (replicative) senescence [1], the contribution or even relevance of this phenomenon to organismal aging has been a subject for continuous debates [2-5]. Although the question still remains open, an increasing amount of evidence, especially from recent years, indicates that cellular senescence (CS) could have a role in aging and age-related diseases (ARDs), rather than being just a laboratory phenomenon [3, 6-11]. In fact, the current situation in the field could be defined as an attempt to understand to what extent and how is CS involved in aging and ARDs.

Apart from an irreversible growth arrest (“Hayflick's limit” — a finite number of cell divisions), the CS phenotype is characterized by cell hypertrophy, an increased metabolic activity including synthesis of macromolecules (RNA, protein, lipid) and organelles [12, 13], increased secretion of pro-inflammatory substances and resistance to apoptosis [7, 8, 11]. After being initially discovered in primary cultures of human fibroblasts, CS has also been found in other cell types such as keratinocytes, endothelial cells, lymphocytes, adrenocortical cells, vascular smooth muscle cells, chondrocytes, etc., both for *in vitro* and *in vivo* conditions [3, 11, 14, 15], and in cell cultures derived from many other organisms examined thus far (e.g., mice, monkeys, chickens, Galapagos tortoise, etc.) [16-18]. Moreover, it appears that CS is not restricted only to dividing cells. At least some features of CS were also found in classical post-mitotic cells such as neurons, myocardiocytes and adipocytes (reviewed by Tchkonia et al. [19]).

The complex nature of aging and aging-associated phenomena including CS requires a holistic view with a focus on the interplay between their components [11, 12, 20, 21]. Here we consider the potential molecular links between CS, longevity, ARDs, oxidative stress, and chronic inflammation from a systems biology perspective. Highlighting the common genes, interactions, regulatory molecules (miRNAs) and common pathways may help in understanding how CS interplays with and contributes to other aging-associated conditions.

## RESULTS AND DISCUSSION

### 1. CS genes share common features with LAGs and ARD genes

A comprehensive data mining of scientific literature brought about a list of 262 human genes identified as being associated with CS (see Suppl. Table 1). These genes possess diverse functions, with the majority falling into three categories: regulation of cell cycle and proliferation, biosynthesis and programmed cell death (for GO functional analysis, see Suppl. Table 2). We have previously shown that longevity-associated (LAGs) and ARD genes also show functional diversity. Besides that, they display a number of distinct features including higher connectivity and interconnectivity, evolutionary conservation, and essentiality to growth and development [22-24]. This combination makes many of them putative candidates as antagonistic pleiotropy genes, i.e., genes which may have undesirable effects later in life, potentially linking aging, longevity and ARDs [25, 26]. Therefore, one of the first questions that arise in this context is whether CS genes share any common features with LAGs and ARD genes.

#### 1.1. Connectivity and interconnectivity: miRNA-regulated PPI networks

**Figure 1:**
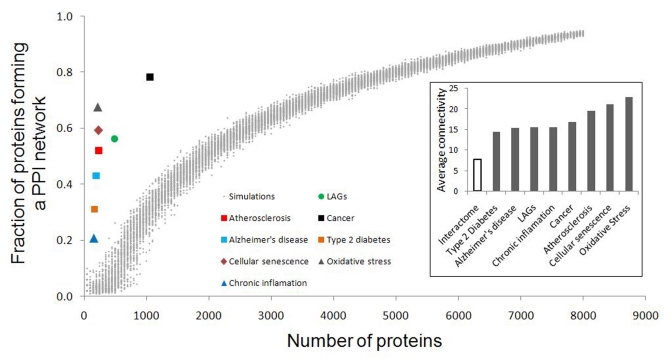
Fraction of CS, longevity and ARD proteins forming a continuous PPI network. Values obtained from simulations with sets of randomly selected proteins are presented as dots. For all the sets of interest, the fraction of interconnected proteins was significantly higher than expected by chance (p < E-25). Insert: average connectivity (number of first-order protein partners) of the sets analyzed in this study. For more details, see Materials and Methods.

To what extent are the CS genes/proteins working in a cooperative manner? In most cases, proteins do not act on their own but rather together with their partners through protein-protein interactions (PPIs). Currently, the human interactome includes approximately 10,000 genes with more than 35,000 physical PPIs ([27], http://thebiogrid.org). Most of CS genes (231 of the 262) as well as LAGs and ARD genes can be found in the human interactome [24]. As shown in Fig. 1 (insert), they have a much higher average connectivity (number of first-order protein partners) compared to all interactome proteins. This is in accordance with observations demonstrating that disease proteins have higher average connectivity than other proteins, and that highly connected proteins are more likely to be disease-associated [28]. It was particularly evident for cancer genes [22] and for genes common to major human ARDs [23, 25].

Not only are CS genes, LAGs, and ARD genes more connected, but they are also highly interconnected. Indeed, when compared with randomly generated sets, the above genes display a significantly higher interconnectivity (the fraction of genes that form a continuous network) (Fig. 1).

**Figure 2:**
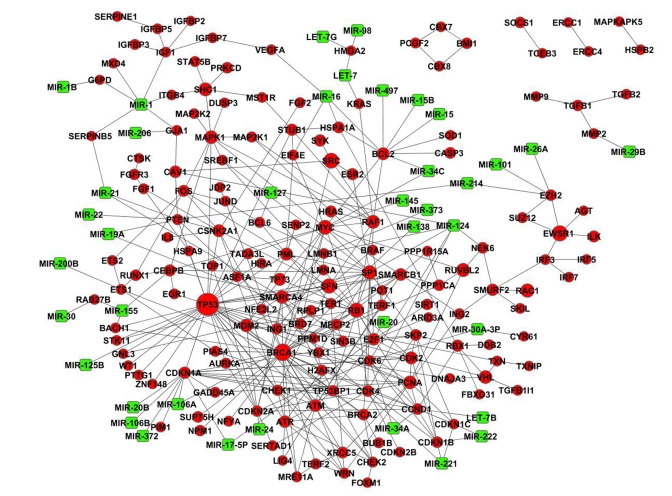
MicroRNA-regulated cellular senescence PPI network. Genes are depicted as red circles and miRNAs as green squares.

For example, 59% of the CS genes are connected between themselves and eventually form a continuous network (Fig. 2), whereas only 4 ± 2% (mean ± SD) genes form a network by chance (p < E−25). The percent of interconnected CS genes would be even higher if other (regulatory) interactions are considered. In particular, if we also take into account post-transcriptional regulation of gene expression by microRNAs (miRNAs), almost two thirds (64%) of the CS genes become connected, either through PPIs or common miRNAs (Fig. 2). Thus, CS genes together with their regulatory miRNAs might work in a cooperative manner by forming a miRNA-regulated PPI network. Such networks are also formed by LAGs and ARD genes (currently available in the NetAge database: [24], http://www.netage-project.org).

#### 1.2. Essentiality

**Figure 3:**
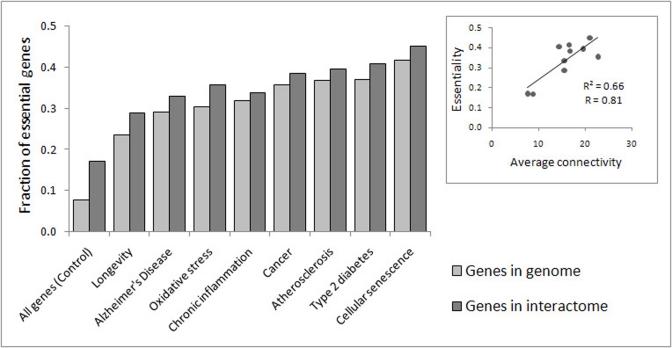
Fraction of genes which are essential to growth and development in each of the gene sets under analysis. The difference between each set and all genes (control) was highly significant (p < E-25). Insert: The correlation between essentiality and average connectivity (R – Pearson's coefficient of correlation; p = 0.004).

Genes with multiple PPIs have a higher probability to be essential, just because the deletions of these genes may result in the disruption of function of a larger number of proteins [28-30]. In line with this assumption, the portion of essential genes among the CS genes, LAGs and ARD genes is much higher than that in the whole genome or interactome (Fig. 3). Moreover, there is a significant correlation between connectivity and essentiality of different sets examined in this study (Fig. 3, insert). It is important to stress that many genes essential for development and growth tend to have detrimental effects at the later stages of life as suggested by the theory of antagonistic pleiotropy [31].

Remarkably, the percent of essential CS genes (42%) is considerably higher than that for all genes (p < E-25) and even higher than the percent of essential LAGs (p = 2.5E-10 and p = 6.2E-06 for entire genome and interactome, respectively), genes involved in Alzheimer's disease (p = 1.5E-04 and p = 0.001) and aging-associated processes (p < 0.002) (Fig. 3). This could at least in part be explained by the fact that the CS genes are highly enriched with genes involved in the regulation of basic, housekeeping processes such as cell cycle, cell growth, programmed cell death, DNA repair, and cellular response to stress (Suppl. Table 2).

#### 1.3. Evolutionary conservation

**Figure 4:**
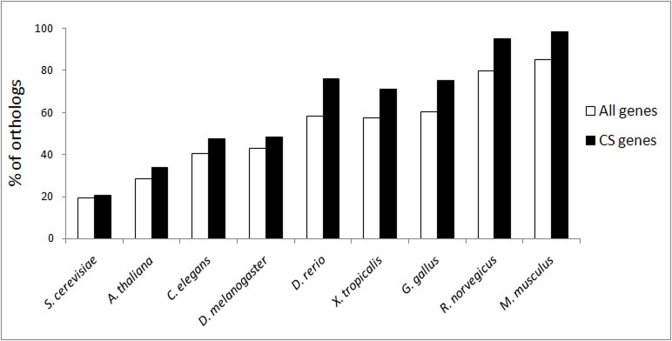
Evolutionary conservation of human CS genes. The difference between CS genes and all genes in InParanoid was significant for *D. rerio* (p = 0.0001), *X. tropicalis* (p = 0.003), G. gallus (p = 0.002), *R. norvegicus* (p = 0.006) and *M. musculus* (p = 0.02).

Essential genes are generally more evolutionary conserved than non-essential ones [32]. In support of this notion are recent findings of Waterhouse et al. [33] who demonstrated that the essential genes from model organisms are significantly enriched in orthologs across the vertebrate, arthropod and fungal lineages. The high percentage of essential CS genes led us to explore the possibility that CS genes are highly evolutionary conserved as a whole. With this in mind, we have examined the frequency of orthologs for the human CS genes in over 100 species found currently in the InParanoid database ([34], http://inparanoid.sbc.su.se). We found that CS genes are significantly more conserved than are the genes in the whole human genome. This is clearly noted across the vertebrate species but the difference is insignificant in lower organisms. In Fig. 4, this is shown for a selected set of well-studied model organisms, from yeast to mouse. The same observation was true for the whole InParanoid set (data not shown). Notably, the pattern of evolutionary conservation of CS genes is almost identical to that of cancer-associated genes (see Fig. 1B in [22]). It would be then tempting to speculate that this similarity is a result of the co-evolution between these two phenomena. Indirectly supporting this assumption are observations on the naked mole rat, a species with an extraordinary resistance to cancer [35], whose fibroblasts do not undergo CS. Instead, they display early contact inhibition, an anti-cancer mechanism based on cell division arrest before reaching a high cell density [36]. Another example is chinchilla, a rodent with a low cancer incidence [37] that also does not develop CS, but evolved other anti-cancer adaptations such as continuous slow cell proliferation [18].

### 2. Molecular links between CS genes, LAGs and ARD genes

For a more specific analysis, we further addressed the following questions: (i) Are there genes common for CS and other aging/longevity-related categories? (ii) Could genes involved in CS, ARDs and in the control of life span interact via direct PPIs or their common partners? (iii) Is the set of CS genes enriched in genes associated with ARDs and age-related conditions? (iv) Could the expression of these genes be under the control of common regulatory molecules, more specifically, miRNAs? (v) Are there common pathways for CS, ARDs and aging-associated processes?

#### 2.1. Common genes

The analysis revealed that 19% of the CS genes are also orthologous to LAGs from model organisms, and 53% of the CS genes are involved in at least one ARD (Table 1). The highest overlap was observed for cancer (53%); lesser values were observed for atherosclerosis (20%), Alzheimer's disease (9%) and type 2 diabetes (9%). The overlap of CS genes with oxidative stress and chronic inflammation associated genes reached 21% and 8%, respectively. In all the above cases, the overlap was significantly greater than expected by chance (p < E-25). Notably, among overlapping genes there are many which are essential for growth and development (from 38% to 55%, depending on the gene set). This high percentage of essential genes is comparable to that found in the Common Gene Signature of longevity and major ARD networks [25].

**Table 1:** Molecular links between CS, longevity, ARDs and aging-associated processes

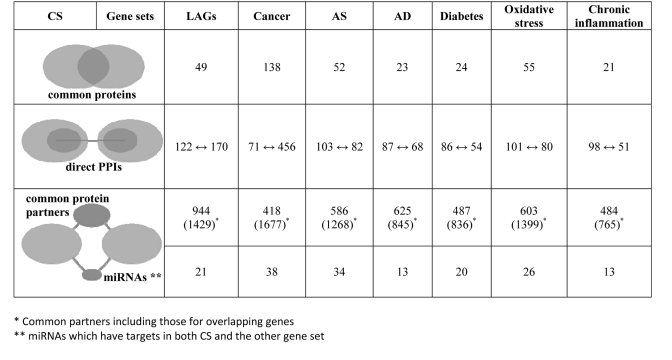

AS – Antherosclerosis, AD – Alzheimer's disease

#### 2.2. Protein-protein interactions and common protein partners

Apart from common genes, a great number of CS proteins directly interact with LAGs and ARD proteins through PPIs (Table 1). As such, the majority of CS genes fall either in the category of common genes or in that of genes directly connected to LAGs or ARD genes. In total, the genes in these two categories exceed 80% of the entire CS set. In addition, there are many common external protein partners, the number of which is more than one order of magnitude higher than that of common genes. As a result, almost all CS genes are linked to longevity and/or ARDs in one of the following ways - as common molecules, by forming protein complexes via PPIs, or through common partners.

#### 2.3. Common miRNA regulators

Another important possibility by which CS genes, LAGs and ARD genes could be linked is the post-transcriptional co-regulation of their expression through common miRNAs. Among the CS genes, 40 have thus far been experimentally validated as being the targets of 39 miRNAs. Of these miRNAs, almost all have targets reported to be involved in cancer and atherosclerosis and many have targets associated with other age-related conditions and longevity (Table 1). Notably, a large number of these miRNAs were indeed found to be directly involved in ARDs (Table 2). For example, out of the 38 miRNAs regulating the expression of both CS and cancer genes, miRNAs belonging to the miR-17, miR-19, miR-21, miR-24, miR-155, miR-214, miR-221, miR-372, and miR-373 families are oncogenic (oncomirs), while miRNAs of the let-7, miR-1, miR-8, miR-15, miR-29, miR-34, miR-101, miR-124, miR-125, miR-127, miR-145 families have a tumor suppressor activity [38, 39, 40, 41]. In fact, as seen in Table 2, all the miRNAs common to CS and atherosclerosis, AD or type 2 diabetes are also cancer-associated.

**Table 2:** Families of common miRNAs reported as being involved in CS and ARDs

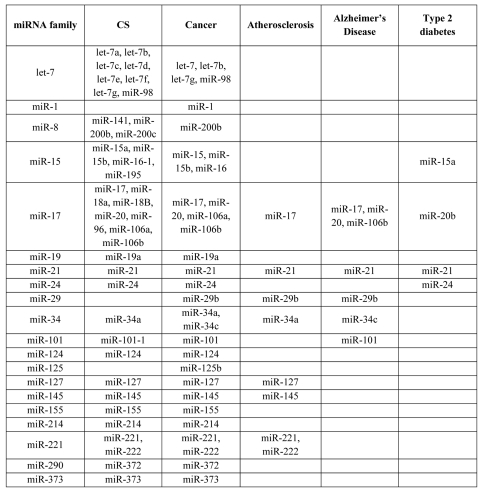

#### 2.4. Cancer genes and miRNAs bridge CS with other ARDs

**Figure 5:**
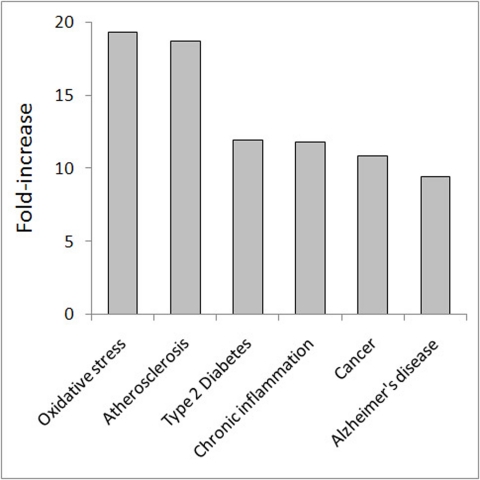
Enrichment of genes involved in ARDs and aging-associated conditions among CS genes. The fold-increase was computed as the ratio between the number of observed genes vs. the expected value. In all cases, the fold-increase was highly significant (p < E-25).

There are many common genes for the major human ARDs [22, 23, 25]. What are these genes and how do they contribute to the overlaps between CS and ARD genes? As already mentioned (see section 2.1), the CS genes are highly over-represented among LAGs and in all major ARDs and aging-associated conditions (Table 1). This also follows from the enrichment analysis as shown in Fig. 5.

The highest fold-increase (19.3-fold vs. the expected value; p < E-25) was found for oxidative stress. This was quite expected as oxidative stress is one of the major CS inducers [42, 43]. The unexpected observation, however, was that the second highest fold-increase value was found for atherosclerosis (18.7-fold; p < E-25), which is almost twice as high as that for cancer (10.9-fold; p < E-25) and other ARDs (10-12-fold; p < E-25). However, further analysis revealed that cancer genes are the primary determinants of the links between CS and ARDs. Indeed, when the cancer genes were removed from the other ARD sets, no significant enrichment was found for any of these sets in CS. In contrast, after the removal of atherosclerosis, diabetes or Alzheimer's disease genes from the cancer set, the enrichment value for cancer genes in CS remained almost unchanged. Thus, the cancer genes are central in linking CS with other ARDs. Of note, the enrichment for CS genes with cancer genes increases when they are also represented in another or several other ARDs (Table 3). In particular, this can explain the high fold-increase in the case of atherosclerosis, since almost all atherosclerosis genes in CS are also cancer-associated (51 of 52). Though the impact of CS on the development of other ARDs is only beginning to be unveiled, accumulating evidence suggests a role of vascular cell senescence in atherosclerosis [44] and a clear cellular senescence component in type 2 diabetes [45] and Alzheimer's disease [46]. Altogether, our findings indicate that (i) cancer genes (together with miRNAs) determine the links between CS and other major human ARDs, and (ii) CS is particularly enriched in genes which are common to both cancer and other ARD(s).

**Table 3:** Enrichment of different sets of ARD genes in CS genes

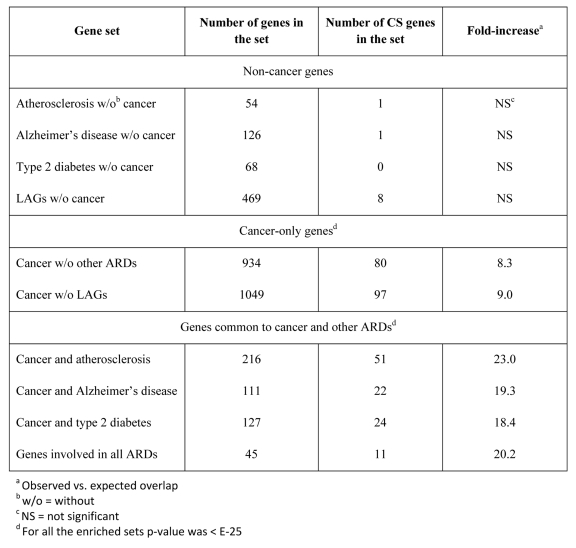

### 3. Common signaling pathways

**Figure 6:**
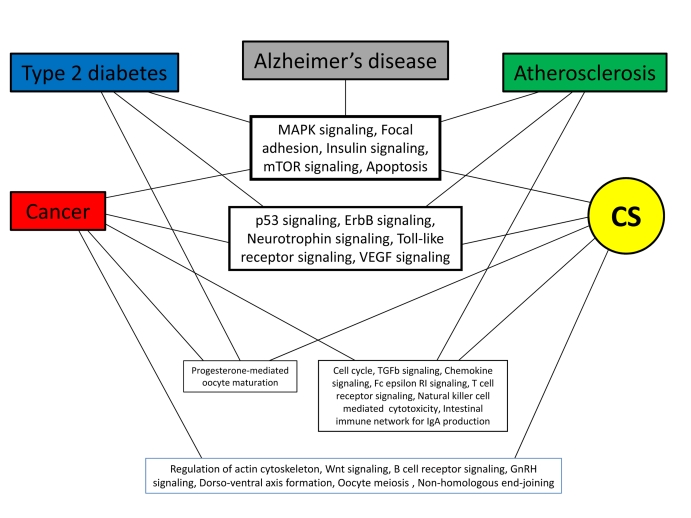
Common pathways enriched across CS and ARDs. Pathways directly involved in specific pathologies were excluded in order to remove bias. See also Suppl. Table 3.

While analyzing the PPI networks of longevity and major human ARDs, we found that about half of the common proteins are related to signal transduction [23]. Moreover, we showed that the vast majority of these proteins are hubs, thus playing a central role in linking different ARDs. Therefore, our next question was whether there are common signaling pathways to CS and other sets examined in this study. Enrichment analysis could serve as a tool for answering this question. We found that several pathways are particularly enriched across CS, ARDs and aging-associated processes (Suppl. Table 3). Surprisingly, among them are many growth-promoting pathways such as the MAPK signaling, insulin signaling, mTOR signaling, ErbB signaling, neurotrophin signalling, etc (Fig. 6). This might seem paradoxical, since an irreversible growth arrest is the major (though not the only) feature of CS. However, recent findings shed light on this apparent discrepancy, clearly demonstrating that stimulation of cells, which have ceased proliferation, with growth-promoting mediators induces CS. In other words, growth-promoting pathways convert reversible quiescence into senescence [12, 47, 48]. Such an activation eventually leads to an enhanced secretion of cytokines, chemokines, proteases and ROS by senescent cells [49], collectively termed as the senescence-associated secretory phenotype (SASP) [9, 50]. This could be especially relevant to aging organisms since they display a persistent activation of growth-promoting pathways [13, 51]. As such, senescent cells are likely to create a pro-inflammatory/tumorigenic microenvironment which promotes the development of aging-associated conditions (chronic inflammation and oxidative stress) and ARDs [6, 51, 53]. In addition, the formation of the “senescent” microenvironment could be greatly attributed to the pathways ensuring cell-cell and cell-ECM (extracellular matrix) interactions, such as “Focal adhesion” and “Regulation of actin cytoskeleton”, which we also found to be significantly enriched across CS and ARDs (Fig. 6, Suppl. Table 3). In particular, the importance of these pathways in CS is highlighted by the fact that the downregulation of caveolin-1, a central regulator of focal adhesion kinase activity and actin stress fiber formation [54] resulted in the re-entry of senescent human fibroblasts into cell cycle and restoration of their clonogenic potential [55]. Remarkably, focal adhesion is among the most enriched pathways in the common signaling network for the major human ARDs and longevity [23].

Given the over-representation of growth-promoting and cell-cell/ECM contact pathways in CS, it was expected to see cancer-associated pathways in the enrichment list, since cell proliferation and growth promotion are intrinsic properties of the cancer cells. Indeed, the most over-represented category in CS is “Pathways in cancer” followed by the pathways for specific forms of cancer, including “Bladder cancer”, “Prostate cancer”, “Colorectal cancer”, “Chronic myeloid leukemia”, “Glioma”, “Melanoma”, and others and tumor suppressor “p53 signaling” (Suppl. Table 3). It should however be kept in mind that the final outcome (CS or cancer) of activation or inhibition of these pathways depends on many additional factors, discussed in details elsewhere [21].

All the above mentioned signaling pathways are also involved in cellular response to stress, further linking CS with aging and ARDs. For example, the CS-associated Gadd45 proteins, prominent stress sensors, are involved in the determination of the stressed cell fate via interactions with p53, MAPK, mTOR and other growth-promoting signaling pathways (reviewed by Moskalev et al. [56]). An age-related decrease in Gadd45 inducibility could promote tumorigenesis, immune disorders and insulin resistance [56]. Depending on the severity of stress and activated gene modules, the CS-associated proteins could either mediate DNA repair with subsequent cell survival (quiescence or re-entry to cell cycle), or induce CS or apoptosis (for details see: [57]). A common event in the stressed cells is a down-regulation of insulin/IGF1-Akt-mTOR axis by up-regulated p53 [58-64]. The resultant temporary quiescence period allows the repair of the DNA damage and the return to the routine cell cycle. However, if the level of stress is too high and/or its duration is too long, the cell cycle arrest turns into CS or apoptosis [64-67]. The induction of CS is mediated by the reactivation of the initially repressed PI3K/Akt-mTOR pathway [68, 69]. Of note, its activation is also considered a hallmark of organismal aging [8, 70, 71]. Accordingly, the inhibition of mTOR signaling with rapamycin decreased the hypertrophic phenotype of senescent cells *in vitro* [47, 72], extended the lifespan and delayed cancer in mice, even when the treatment was initiated later in life [73]. Thus, the common signaling pathways and the very mechanisms of CS induction link it to aging and ARDs.

## CONCLUDING REMARKS

Our study shows that CS is tightly interconnected to aging, longevity and ARDs, either by sharing common genes and regulators or by PPIs and eventually by common pathways. The identification of a common molecular basis is an important step towards understanding the relationships between all these conditions. The next natural step would be the integration of these data with gene/miRNA expression profiles. Such integration could further highlight the key players in linking CS and ARDs. However, this is not a trivial task as the vast majority of data concerning CS derives from *in vitro* studies on fibroblasts while ARDs are well studied in a variety of cells and *in vivo* systems. Broadening the CS investigation by including more cell types, 3D *in vitro* models and *in vivo* studies will help in developing a more holistic view on the CS phenomenon.

Our analysis also provides the basis for new predictions since the genes common to both cancer and other ARD(s) are highly likely candidates to be involved in CS and *vice versa*. In addition, a higher connectivity, evolutionary conservation among vertebrates and essentiality may increase the probability for CS genes to be found as being involved in ARD(s).

Another interesting finding is the similarity between the patterns of evolutionary conservation of CS and cancer genes, which suggests the co-evolution of these two phenomena and calls for a wide comparative study. Of special interest would be the investigation of CS in species with exceptional longevity and resistance to tumors, such as bowhead whales. The comparative studies could shed more light on the links between cancer and CS, and may help in understanding why cancer genes stand at the crossroad of CS and ARDs.

An important point for future investigations is examining the role of CS in the formation of an aging microenvironment and its impact on the pathogenesis of ARDs. In turn, the CS phenotype could be modulated by microenvironment. As demonstrated by Choi et al. [74], the interaction of senescent cells with ECM from young cells is sufficient to restore their replicative potential and youthful morphotype. Our present and previous [23] *in silico* analyses together with experimental data [55] indicate that a special emphasis should be put on focal adhesion and its interactions with growth-promoting pathways.

The principal questions remain as to when it is worthwhile to induce and when to inhibit CS. What strategy is preferable, anti- or pro-CS? These questions stem, in particular, from the suggested antagonistic nature of CS in cancer [6, 75]. The same dual role of CS could also be true for other ARDs but yet has to be established. Whatever the case, it seems quite plausible that the links between CS and ARDs may vary at different stages of disease and life. Induction of CS was proposed as a potential anti-cancer therapy [76, 77]. Alternatively, the recent study of Baker et al. [10] showed that the drug-induced clearance of senescent cells from an early age delayed the onset of several age-related conditions such as sarcopenia, cataracts and loss of adipose tissue in progeroid mice, and these beneficial effects were also pronounced when the elimination of the senescent cells was initiated in the adults. As a feasible tool for enhancing the clearance of senescent cells, Krizhanovsky et al. [78] suggested immunostimulatory therapy. To some extent, in favor of the anti-CS strategy are the results of our preliminary analysis hinting that the pro-longevity genes are rather anti-CS while the pro-CS genes dominate among the anti-longevity genes (unpublished data). Given the potential benefits of the anti-CS approach, an intriguing possibility could be based on iPS technology. Using this technology, Lapasset et al. [79] demonstrated that senescent cells and the cells derived from centenarians could be reprogrammed and eventually rejuvenated. As a perspective for an *in vivo* application of this method, “a possible scenario may be that after several rounds of iP, the microenvironment itself would also assume a younger phenotype” [80]. Future studies of the different aspects of the links between CS and ARDs will help in selecting the most adequate therapeutic strategy.

## MATERIALS AND METHODS

A list of genes that have been established as being involved in CS was compiled from scientific literature and manually curated. The selection of genes was based on two lines of evidence: 1) genetic or RNA interference (RNAi) interventions (gene knockout, partial or full loss-of-function mutations, RNAi-induced gene silencing, overexpression) which reportedly cause cells to either induce, inhibit or reverse CS, and 2) genes shown to be markers of CS. The lists of LAGs and the genes involved in ARDs and aging-associated processes (oxidative stress and chronic inflammation) were obtained from databases and scientific literature as described in detail elsewhere [23, 24, 81]. The list of differentially expressed miRNAs in CS and of those which have been shown to affect CS was gathered and manually curated from the scientific literature [82-86]. Annotations regarding the involvement of miRNAs in different ARDs were taken from the Human MicroRNA Disease Database ([87], http://cmbi.bjmu.cn/hmdd). Oncogene/Tumor Suppressor classification of cancer-associated miRNAs was done according to Wang et al. [38]. Experimentally validated targets of miRNAs have been obtained from the TarBase database ([88], http://diana.cslab.ece.ntua.gr/tarbase/) and updated by data mining from the scientific literature.

Evolutionary conservation of CS genes was analyzed using the InParanoid7 – Eukaryotic Ortholog Groups database ([34], http://inparanoid.sbc.su.se). To exclude inparalogs, we have used the default threshold score of 0.05. Essentiality of human genes was evaluated based on the data on mouse lethal phenotypes, which were retrieved from the Mouse Genome Informatics database ([89], www.informatics.jax.org). Functional and pathway analyses were performed with the tools provided by DAVID Bioinformatics Resources 6.7 ([90, 91], http://david.abcc.ncifcrf.gov) using data from the KEGG database ([92], http://www.genome.jp/kegg/pathway.html) and Gene Ontology ([93], http://www.geneontology.org). Protein-protein interacttion (PPI) data from the BioGRID database ([27], http://thebiogrid.org), human interactome release 3.1.71, was used for the analysis of connectivity and interconnectivity. The largest fraction of proteins which forms a continuous network was used as a measure of interconnectivity. To generate control data, simulations with sets of randomly selected proteins from the interactome were performed. The size of protein sets ranged from 50 to 8000 genes, with a step of 50. In each case, one hundred simulations were run and the relation between the size of the set and the fraction of genes interconnected by chance was quantified. The simulations and the creation of the microRNA-regulated PPI CS network were done using YABNA (Yet Another Biological Networks Analyzer). The YABNA software program and the algorithm for the construction of miRNA-regulated PPI networks have been previously described in detail ([24, 25], http://www.netage-project.org). The graphical output of the CS miRNA-regulated PPI network was generated using Cytoscape 2.8.0 ([94], http://www.cytoscape.org/).

The statistical package for the social sciences (SPSS, Inc., Chicago, IL) software was used for the statistical evaluation of the results. Significance of the difference between mean values was calculated using the Student's t-test. The difference between observed and expected values was evaluated using the chi-square test. Pearson's coefficient of correlation was used for pair-wise correlative analysis. Differences were considered significant at p-value less than 0.05. Statistical analysis of the enrichment of GO categories and KEGG pathways was carried out using DAVID Bioinformatics Resources 6.7 ([90, 91], http://david.abcc.ncifcrf.gov), with Bonferroni correction.

## SUPPLEMENTAL TABLES

Supplementary Table 1

Supplementary Table 2

Supplementary Table 3
